# Urdu Nasta’liq text recognition using implicit segmentation based on multi-dimensional long short term memory neural networks

**DOI:** 10.1186/s40064-016-3442-4

**Published:** 2016-11-25

**Authors:** Saeeda Naz, Arif Iqbal Umar, Riaz Ahmed, Muhammad Imran Razzak, Sheikh Faisal Rashid, Faisal Shafait

**Affiliations:** 1 National University of Sciences and Technology (NUST), Islamabad, Pakistan; 2 Department of Information Technology, Hazara University, Mansehra, Pakistan; 3 German Research Center for Artificial Intelligence (DFKI), Kaiserslautern, Germany; 4 King Saud Bin Abdul Aziz University for Health Sciences, Riyadh, Saudi Arabia; 5 University of Engineering and Technology (UET), Lahore, Pakistan

**Keywords:** Urdu OCR, BLSTM, MDLSTM, CTC

## Abstract

The recognition of Arabic script and its derivatives such as Urdu, Persian, Pashto etc. is a difficult task due to complexity of this script. Particularly, Urdu text recognition is more difficult due to its Nasta’liq writing style. Nasta’liq writing style inherits complex calligraphic nature, which presents major issues to recognition of Urdu text owing to diagonality in writing, high cursiveness, context sensitivity and overlapping of characters. Therefore, the work done for recognition of Arabic script cannot be directly applied to Urdu recognition. We present Multi-dimensional Long Short Term Memory (MDLSTM) Recurrent Neural Networks with an output layer designed for sequence labeling for recognition of printed Urdu text-lines written in the Nasta’liq writing style. Experiments show that MDLSTM attained a recognition accuracy of 98% for the unconstrained Urdu Nasta’liq printed text, which significantly outperforms the state-of-the-art techniques.

## Background

The tremendous advances in the field of image processing and computational intelligence have resulted in a significant progress in the development of character recognition applications for complex scripts. Particularly, several OCR systems have been developed in the commercial as well as open source domain for the recognition of Asian scripts like Chinese, Japanese, and Korean; such as ABBYY FineReader,^1^ MeOCR,^2^ JOCR^3^ and Tesseract (Smith 2007). However, progress in the recognition of Arabic script has been relatively slow mainly due to the special cursive characteristics of the script. Recognition of its derivative scripts like Nasta’liq is further complicated due to its calligraphic nature (Naz et al. 2014a). We point out these complexities to show that the work done for Arabic script recognition is not suitable for Urdu Nasta’liq (cf. “Urdu–Nasta’liq script” section) script.

To handle these complexity in Arabic script in general, and in Urdu Nasta’liq script in particular, a number of different approaches have been studied (Naz et al. 2014a). These approaches can be primarily categorized under Analytical and Holistic frameworks. Analytical approaches are further divided into explicit segmentation and implicit segmentation based methods. Explicit segmentation approaches usually have three major steps: over-segmentation, grouping, and classification. In the first phase, the ligature is segmented into units not bigger than various shapes of character and then grouping is performed onto the recognized unit to form ligature hypotheses. These ligature candidates are then fed to the recognition engine to find the most plausible combination. These approaches are script dependent and are mainly based on the analytical characteristics of the particular script to perform segmentation (Naz et al. 2015b). Accurate and consistent segmentation under various document degradations usually becomes a performance bottle-neck of such systems. Implicit segmentation approaches are based on predefined labels or code-books for images of text-lines, words or ligatures. The labels with their corresponding images are fed to a given machine learning model, which is then used to identify segmentation cue points at recognition time without pre-segmented units of ligatures (Saeed and Albakoor 2009). On the other hand, holistic approaches deal with the shapes of the entire ligatures. In this way, the shape of the ligature or sub-word is learned by the model without segmenting it into sub units. In holistic approaches, the system is trained for recognizing each ligature/word directly. Holistic approaches are considered to be script independent. However, they suffer from scalability issues as the number of unique shapes regarding ligatures or sub-word in a particular script may be very large. Urdu has more than 25,000 ligatures (Lehal 2012), thus the holistic based approaches are not suitable for such a large number of classes. However, small scale applications such as city names, bank checks etc, with limited vocabulary could be developed using holistic approaches.

Urdu Nasta’liq writing style has a diagonal nature (the pen stroke not only moves from right to left, but also from top to bottom). Therefore, we need such a model, which not only learns patterns/sequences from right to left and from left to right, but also from top to bottom and from bottom to top. Therefore, in this work we are proposing the adaptation of Multidimensional Long Short Term Memory (MDLSTM) neural networks for the recognition of Urdu Nasta’liq script, under the implicit segmentation approach. The reason of choosing MDLSTM is that it can scan the input image in all four directions (up, down, right and left). The MDLSTM is one of the variants of Recurrent Neural Network (RNN) and is effectively used for multi-dimensional sequence learning (Naz et al. 2013b). The novelty of this work in general is the use of MDLSTM for the first time for the Urdu Nasta’liq script recognition and particularly to investigate MDLSTM architecture against the diagonal nature of Nasta’liq script. Furthermore, we are also proposing the use of Connectionist Temporal Classification (CTC) layer as an output layer. CTC can probabilistically align the labels against the learned sequences in the image, thus avoiding explicit segmentation. To evaluate the performance of MDLSTM against Urdu Nasta’liq script, we have used Urdu Printed Text Images (UPTI) dataset. This dataset has 10,000 text-lines written in Urdu Nasta’liq writing style.

The rest of this paper is organized as follows: “Urdu–Nasta’liq script” section illustrates the complexities of Urdu script. “Related work” section and “Database” section describe the related work and dataset. “Methods” section presents MDLSTM based Urdu Nasta’liq recognition system and finally “Conclusion” section presents conclusions of the work.

## Urdu–Nasta’liq script

**Fig. 1:**
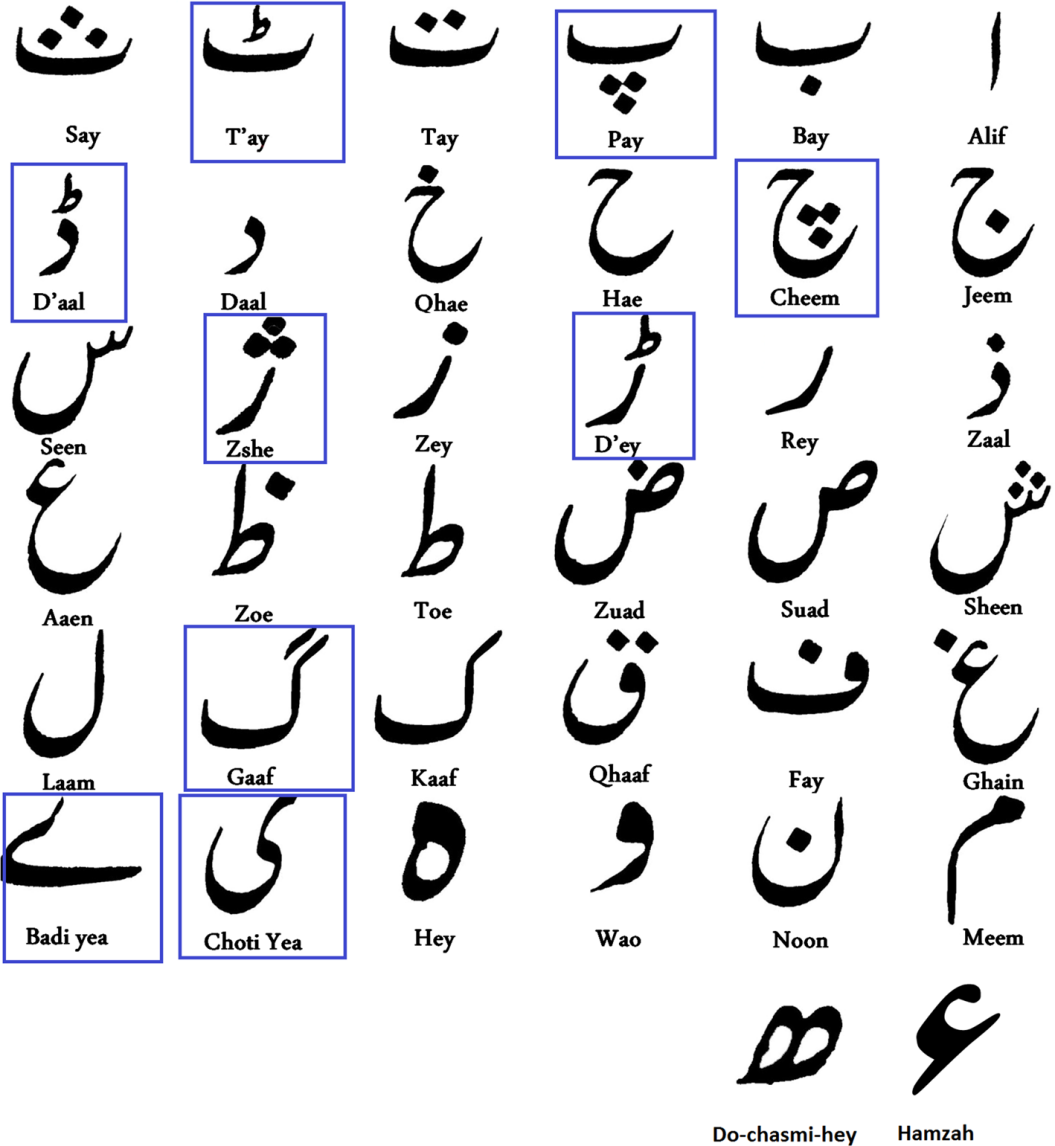
Basic 38 alphabets of Urdu. The *blue rectangles* show the additional characters to represent new sounds present in Urdu language

Arabic and its derivative languages share the same basic writing script; however the alphabet of the derivative languages is extended to deal with sounds that are particular to the local languages. Such new characters in a derivative Urdu language are shown in blue rectangle in Fig. 1.

**Fig. 2:**
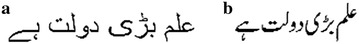
A sentence in: **a** Naskh writing style, **b** Nasta’liq writing style

The development of Arabic calligraphy led to the creation of several decorative writing styles that were designed to accommodate special needs. The most outstanding of these styles are Nasta’liq, Naskh, Koufi, Thuluthi, Diwani and Rouqi. Naskh and Nasta’liq are the two commonly followed styles. Arabic uses Naskh writing style (Fig. 2a) while Urdu and Persian follow Nasta’liq (Fig. 2b). The writing direction is from right to left in all of these scripts.

Nasta’liq script emerged as a combination of two other Arabic scripts Naskh and Talique and gained popularity due to its beauty and compactness. Hence, Nasta’liq script carries the properties of both script and due to the calligraphic nature of this script; it introduces unique challenges that do not occur in Naskh and other Arabic scripts (Naz et al. 2014a, 2015b). These complexities make the character segmentation and recognition in Nasta’liq script a very challenging task.

**Fig. 3:**
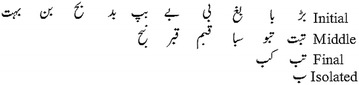
Variation of shapes of Character ’bay’ ( ) at different position

Arabic script and its derivatives classify characters into two groups: joiner and non-joiner. The joiner characters join with their predecessor and successor characters when they occur on the initial, middle or final position in the word. Non-joiner characters, on the other hand, split the word when they occur and hence appear only in the isolated form or the last character of a ligature. For example in Naskh, each individual character has up to four shapes according to its position in the ligature or sub-word (Initial, Middle, Final and Isolated). In contrast, Nasta’liq writing style leads to several different morphologies of the same character rather than four (Akram et al. 2014). The character shape not only depends upon the location but also on the associated characters at both sides. Different shapes of character “
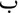
” “bay” are shown in Fig. 3. Thus, this connectivity of characters leads approximately to 26,000 ligatures for Urdu Nasta’liq script (Lehal 2012).

Another complexity is introduced by multiple baselines in the Nasta’liq script (Naz et al. 2014b). The baseline is a virtual line on which characters are combined to form the ligatures and it facilitates both readers and writers. Unlike the Naskh script, character may appear at different descender line depending upon the associated characters. In Nasta’liq writing style, the varying locations of ascenders and descenders leads to errors in the accurate detection of the baseline because of their oblique orientation and long tail. Thus, without prior knowledge of the word and text-line structure it is quite difficult to estimate the baseline.

Due to the calligraphic nature of Nasta’liq script, character segmentation is challenging and prone to error (Hussain and Ali 2015). The purpose of segmentation is to divide the ligature into recognizable units or characters. Segmentation has considerable overheads and it is difficult to find accurate segmentation points for Nasta’liq script (Lehal 2012).

## Related work

In traditional segmentation-based approaches, the performance of character recognition depended on character segmentation accuracy (Naz et al. 2013b). As discussed earlier in “Urdu–Nasta’liq script” section, explicit segmentation of cursive Nasta’liq script is difficult and prone to errors (Naz et al. 2015b). The new trend is diverting towards implicit segmentation as these approaches, especially the ones based on Recurrent Neural Networks (RNN), have shown promising results for cursive scripts. In the following text, we discuss the benchmark results based on implicit segmentation for different languages using RNN classifier.

Graves et al. (2009) applied Bidirectional Long Short-Term Memory (BLSTM) networks for online and offline character recognition on IAM-onDB and IAM-DB databases without explicit segmentation of words into characters and reported word-level recognition accuracies of 79.7 and 74.1%, and character-level accuracies of 88.5 and 81.8% respectively. The experiment showed that BLSTM out-performed the state-of-the-art segmentation based and segmentation free approaches. Graves further extended 1-dimensional (1D) LSTM into two-dimensional (2D) LSTM and presented an MDLSTM system based on a hierarchy of MDRNN and CTC (Graves et al. 2006) in ICDAR-2009 cursive handwriting recognition competition (Mozaffari and Soltanizadeh 2009). They used the raw pixels as input to the MDLSTM classifier and obtained accuracy of 91.85 and 95.9% for Arabic characters and digits respectively. Further, Graves and Schmidhuber (2009) presented another MDLSTM based system (Märgner and El Abed 2009) and achieved the highest results (91.4%) in the ICDAR 2009 competition on IFN/ENIT dataset (Mozaffari et al. 2008). A remarkable contribution of Graves in the field of character and speech recognition is the development of open source library, RNNLIB (Graves 2013), that implements RNNs, BLSTM, and MDLSTM architectures. Rashid et al. (2013) extracted raw pixels from Arabic words and fed them to MDLSTM to achieve 99% recognition on APTI dataset and subsequently win ICDAR 2013 Printed Arabic Recognition Competition.

Recently, Anupama and Sai (2015) implemented BLSTM using raw pixels for Oriya language and claimed 95.85% recognition rate. Another recent contribution (Pham et al. 2013) performed classification based on raw pixels from the text image for English, French and Arabic using MDLSTM classifier. Pam et al. presented the effectiveness of dropout in the traditional RNN architectures and reported 91.1, 85.6 and 90.1% on RIMES (French) (Grosicki et al. 2009), IAM (English) (Marti and Bunke 2002) and OpenHaRT (Arabic) (Morillot et al. 2013b) datasets, respectively. In ICDAR-2015, Chherawala et al. (2013) presented a scale invariant Pashto ligature recognition system using MDLSTM and reported 99% recognition rate. There are also some works using BLSTM or MDLSTM systems based on feature vectors rather than raw pixels (Ahmad et al. 2015; Chherawala et al. 2013; Liwicki et al. 2007).

In the literature of Urdu OCR using implicit segmentation approach, Ul-Hasan et al. (2013) performed two experiments for Urdu text-lines recognition on UPTI database (Ahmed et al. 2016) using one dimensional BLSTM and a sliding window. In the first experiment, they considered the shape variations of Urdu characters (i.e. initial, middle, final and isolated) as separate classes. In the second experiment, they merged all shape variations of one basic character into one class and extracted the raw pixels from a $30 \times 1$ sliding window to train the BLSTM classifier. They achieved character recognition rates as 86.4 and 94.85% for the two experiments respectively. Another work on UPTI dataset is reported in Ahmed et al. (2016), in which Ahmed et al. employed BLSTM on raw pixels for shape variations scenario and without shape variations scenario using a $30 \times 1$ sliding window for Urdu text-lines and reported recognition rate upto 88.4% for the first scenario and 88.94% for the second scenario.

Due to the use of UPTI dataset for Urdu text recognition, we also mention the work of Morillot et al. (2013a). They presented a segmentation free OCR system for recognition of clean as well as degraded ligatures images of Urdu Nasta’liq. They segmented the ligatures from the text-lines and recognized the ligatures based on holistic features. They achieved 88.8% accuracy rates for degraded ligatures and 91% recognition rate for the clean ligatures.

It is mentioned above, that works in Ul-Hasan et al. (2013) and Ahmed et al. (2016) implemented BLSTM for recognition of Urdu Nasta’liq text recognition and statistical features extracted and fed to MDLSTM in Naz et al. (2015a). To the best of our knowledge, MDLSTM approach using raw pixels has not been explored for Urdu Nasta’liq recognition. In the proposed system, we investigate MDLSTM using raw pixels for Urdu Nasta’liq recognition. The description of the database used in our study is given in the following sections.

## Database

Datasets plays a vital role in evaluating the performance of any pattern recognition system. An Urdu Printed Text Image (UPTI) dataset has been developed (Sabbour and Shafait 2013) for research community in the field of Urdu OCR as an analogy to APTI (Arabic Printed Text Image) dataset (Slimane et al. 2009). It consists of various versions to measure accuracy of recognition system on images of text-lines. These versions include degraded text-lines, ligatures/sub-words and degraded ligatures. The synthetic text-lines were collected from Jang newspaper,^4^ which covers different political, social, and religious issues. In our experiment, we worked on text-lines version of UPTI dataset. The dataset contains 10,000 images having Urdu text-lines. These images are further split into training, validation and test sets. As no standard splits are defined in the dataset, we defined our own splits for experiments. The split has been done by making 68% of the images as Training set, and 16% each as validation and test sets respectively. The detailed statistics of the split of the UPTI database and the total number of occurrences of all characters into training, validation and testing sets are shown in Table 1.

**Table 1:** The UPTI dataset splits used in this work

Sets	Text-lines	Characters
Train set	6800	6,35,107
Validation set	1600	1,62,513
Test set	1600	1,73,029
Total	10,000	9,70,649

The total number of instances of each character in training, testing and validation sets

**Fig. 4:**
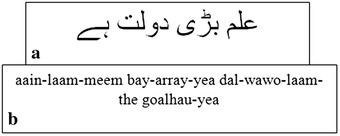
A sentence in Urdu: **a** text line image, **b** ground truth or transcription

Further, for supervised learning it is necessary that the input sequence is well transcribed by its corresponding target labels. Generally, such transcriptions are provided as ground truth data. As MDLSTM is a supervised learning model, it requires the ground truth values for each image in the input space to train the model. An input image and its corresponding ground truth are illustrated in Fig. 4.

**Fig. 5:**
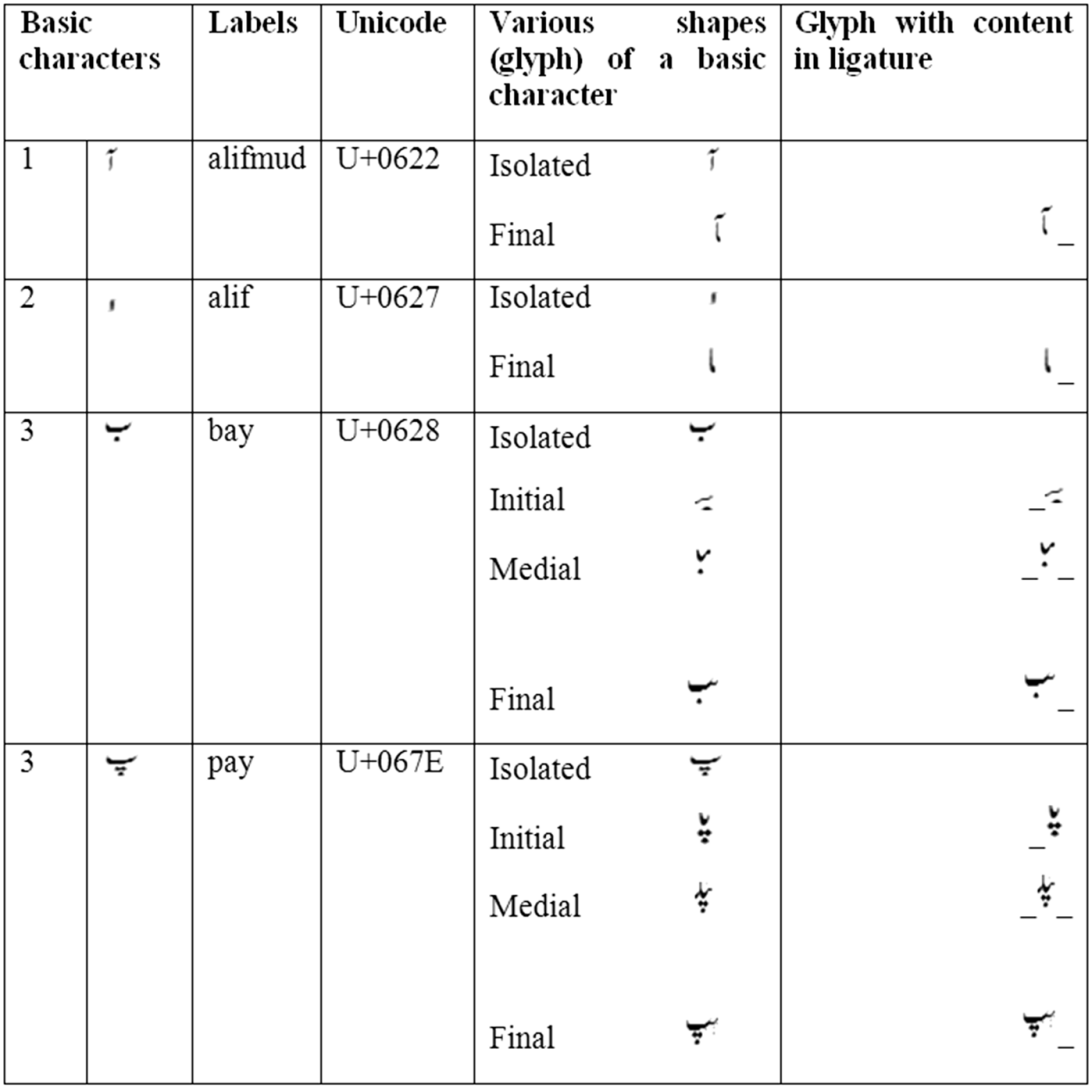
Assignment of labels for creating a single class for various shapes of a basic character. The characters 1 and 2 are example of non-joiner characters having two shapes which split the word into sub-word/ligatures. The characters 3 and 4 are examples of joiner characters having more than 2 shapes which join its preceding or proceeding character. The shape/glyph of a character with content in ligatures is shown in the *last column*

In our experiment, different shapes such as initial, middle, final and isolated form of a basic character are considered as one class and assigned one label as shown in Fig. 5. So, we have 44 labels in total. Forty two unique labels for character level transcription including 38 basic characters (see Fig 1) with extra 4 common characters (noonghuna “
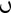
”, wawohamza “
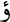
”, haai “
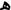
”, and yeahamza “
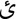
”), one label for ‘SPACE’ and one extra label for the blank (background) are used.

## Methods

In this section, we present the experimental design of Urdu Nasta’liq text line recognition. We adopted pixel based MDLSTM approach reported in Naz et al. (2013b) for recognition of cursive Urdu script. The normalized grayscale text-lines and the corresponding transcriptions are fed to the MDLSTM network. The network is trained on raw pixels of images having Urdu text-lines and the CTC layer is deployed to generate the sequence of labels for the text line images. During recognition, a normalized grayscale test image is classified through the trained network and it generates the text line transcription.

### Preprocessing and features extraction

**Fig. 6:**
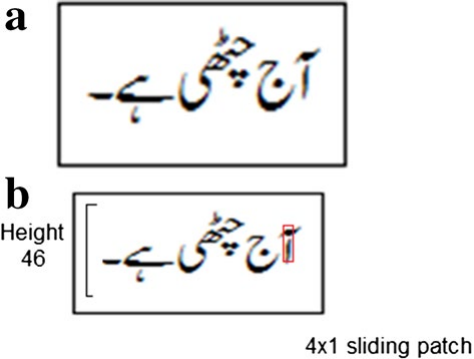
**a** Original image, **b** image converted into gray scale image and re-sized to a height of 46 keeping aspect ratio locked. Then a 4 × 1 sliding patch is used to extract pixel values as the features

The preprocessing stage is essential for pruning unwanted artifacts from the data. Therefore, in the preprocessing stage of our proposed model, the original image of Urdu text-line (see Fig. 6a) is first converted to gray-scale, and then height is normalized by 46 pixels, keeping the aspect ratio locked. After this the resulted text-lines have height to 46 pixels and the width is variable as shown in Fig. 6b. Finally, the text-lines are scanned by using a small patch having a height of 4 pixels and a width of 1 pixel. The extracted raw pixels based features are then passed to the MDLSTM network. The detailed training of the model is explained in the next section.

### Training model

**Fig. 7:**
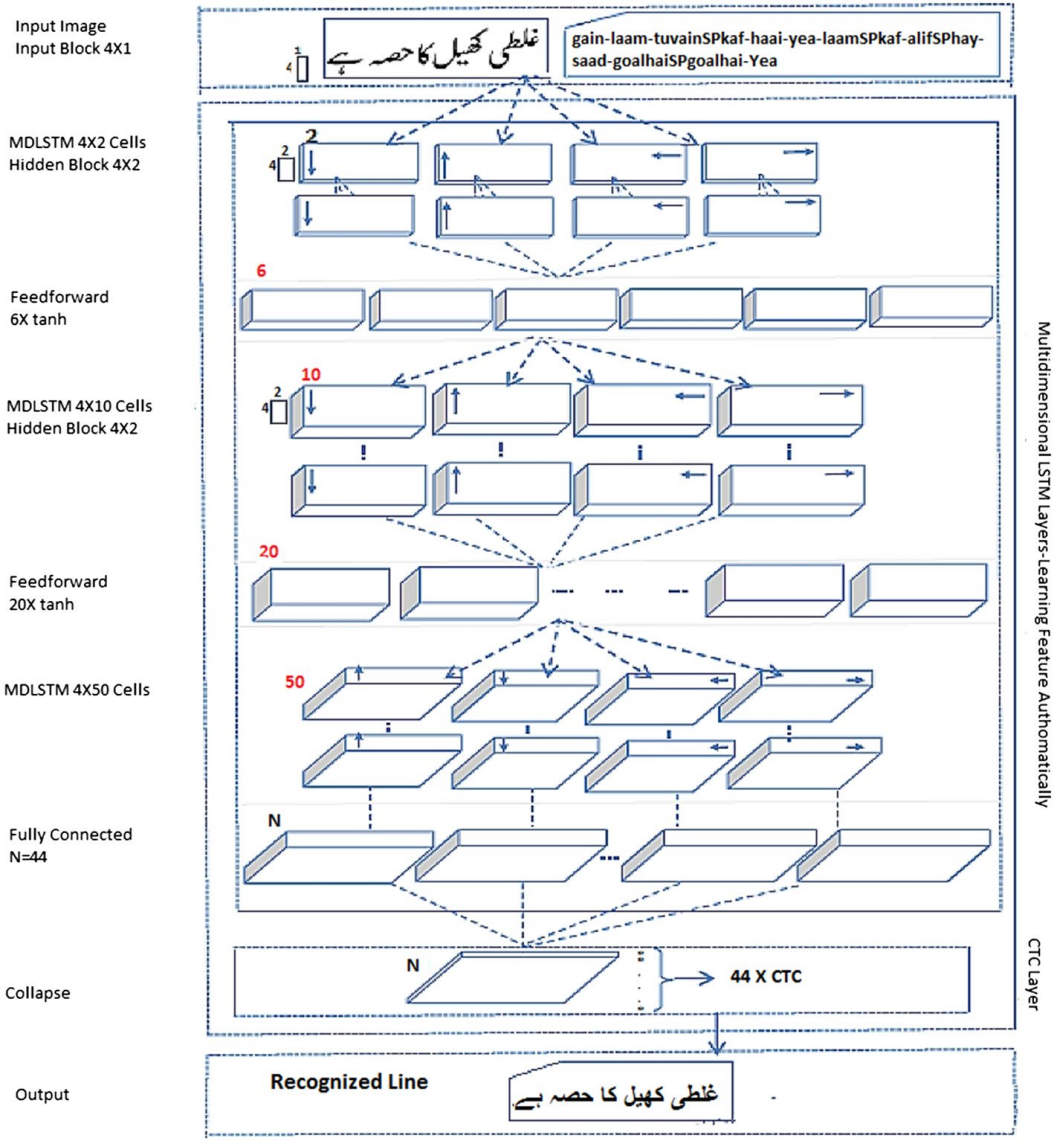
Architecture of MDLSTM for Urdu Nasta’liq text line recognition

The overall architecture of MDLSTM network for recognition of Urdu Nasta’liq text-lines (see Fig. 7) is composed of the input block size, hidden block size, sub sample size and LSTM layer size with the maximum number of nodes for CTC output layer. The input block is the size of small patches that scan the pixels of the image for further processing. The hidden block size is the size of small patches at each hidden layer in the MDLSTM network. The sub-sampling layers are between each pair of hidden layers and the size of the sub-sampling specifies the total number of feed forward $\tanh$ units in the layers of sub-sampling.

#### Network parameters

Different preliminary experiments are performed with different network parameters. The purpose of these preliminary experiments is to choose the network parameters which give comparatively low error rates in a reasonable time. The parameters which are mentioned in Table 2 are the ones we finally select for training the MDLSTM; the other choices of parameters along with their error rates and total training time are given in Table 3. The system is trained for extracting discriminative features form the raw pixels of a text line image. The other parameters are the learning rate ($1 \times 10^{-4}$) and momentum (0.9). The total number of weights of the network cells are 551,405. The training was stopped when there was no improvement in the error rate of validation set for 40 epochs.

**Table 2:** Selected parameters for training the network

Parameters	Values
Input block size	4 × 1
Hidden bolck size	4 × 2
Subsample sizes	6 and 20
Hidden sizes	2, 10, 50
Learn rate	1 × 10^−4^
Momentum	0.9
Total network weight	551,405

**Table 3:** Different parameters for training MDLSTM and the corresponding training and validation error rates

Parameter	Value(s)	Error rate (%) train set/Validation set	Number of passes	Approx. Ave. time per epoch (minutes)
Learning rate	$1\times 10^-3$	0.96/1.98	332	36
	$1\times 10^-4$	0.85/1.83	227	35
	$1\times 10^-5$	99.508/99.65	398 (experiment was terminated)	40
	$1\times 10^-6$	98.67 /98.86	403 (experiment was tenninited)	40
Sub-sampling	6 and 20	0.85/1.83	227	35
	6 and 40	1.73/3.93	256	40
	12 and 40	2.14/3.64	307	30
	24 and 80	0.8/4.47	289	55
Hidden layer sizes	2, 4 and 20	25.88/25.69	251	36
	4, 10 and 30	13.46 /19.20	256	45
	2, 10 and 50	0.85/1.83	227	35
	4, 20 and 100	0.82/1.80	236	75

#### MDLSTM based Urdu character recognition system

After choosing suitable parameters, the image of a text line is processed by dividing it into small patches using input blocks having width of 1 column and height of 4 rows. The raw pixels of the image are collapsed to a vector of length 4 and are fed to the MDLSTM with the corresponding ground truth. The small patches of the image are then scanned through forward and backward passes in all four directions (horizontally and vertically) by MDLSTM to extract and learn distinct features. The detailed schema of implementation of MDLSTM is shown in Fig. 7.

**Fig. 8:**
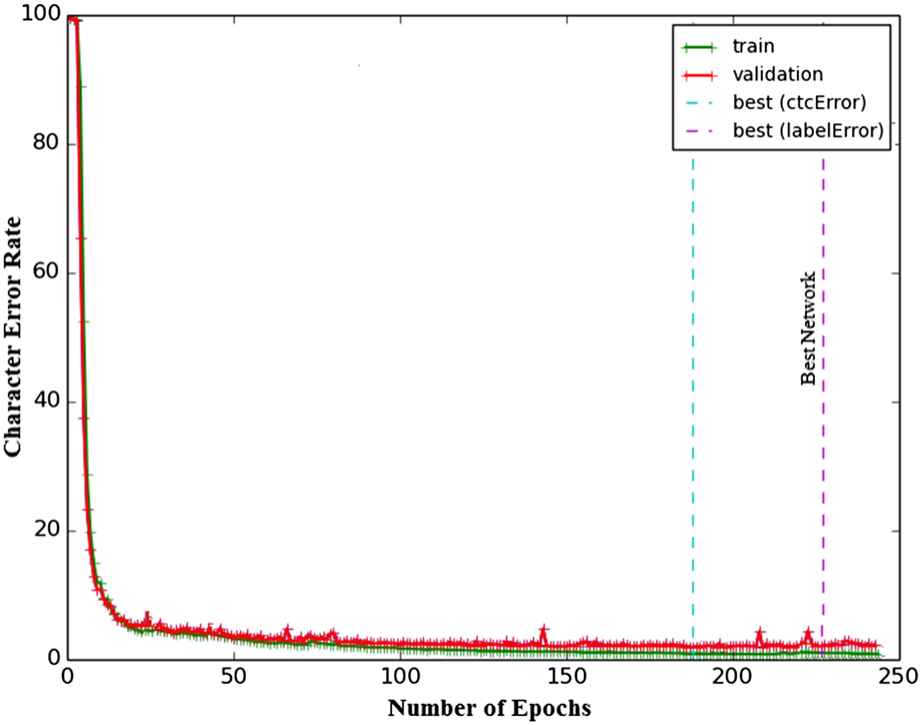
Character error rates on different number of epochs for training the MDLSTM on the training and validation sets. The training network converged to the minimum error rate at epoch number 227

The remaining network layers are described as follows; there are 3 hidden layers consisting of LSTM cells. The size of the each layer is 2, 10 and 50 respectively. The hidden layers are fully connected. These three layers are further separated by two sub-sampling layers. These sub-sampling layers have size of 6 and 20 respectively. The sub-sampling layers are feed-forward $\tanh$ layers. The features are then collected into 4 × 2 hidden blocks. These 4 × 2 blocks are then fed to the layer of feed forward which is using $\tanh$ summation units for the cell activation as shown in Fig. 7. The MDLSTM activation finally collapses into a one dimensional sequence and CTC layer labels the contents of the one dimensional sequence (Fig. 8).

The proposed system based on MDLSTM architecture creates blocks for two purposes. First purpose of the 4 × 1 input block is the collection of local contextual information from the input image and the second purpose of the 4 × 2 hidden block size is to extract discriminative features and to reduce the size of the feature vector to be fed to the activation layer. Since the CTC layer needs one dimensional sequence as an input, therefore the vertical dimension is reduced. The sub-sampling or reduction is done by the hidden layers like in the convolutional neural networks (Table 4).

**Table 4:** Error rates for Urdu Nasta’liq text line recognition for training and validation sets

Errors	Training set (%)	Validating set (%)
Label error	0.85	1.83
Deletions	0.22	0.48
Insertions	0.150	0.22
Substitutions	0.480	1.21
CTC error	3.11	8.28

### Recognition

The test set of 1600 unseen images is fed to the trained MDLSTM model for classification. Once again, each image is converted to gray scale and then its height is normalized to 46 pixels. The classification and recognition of 1600 images has taken a total time of 1 minute and 43.3 seconds on a 3.4 Ghz Intel Core i7 machine with 8 GB RAM. After recognition, the predicted text is generated against each image as the output. Meanwhile, the predicted text is compared against the corresponding ground truth and the overall error rates are calculated.

### Results and discussion

To evaluate the accuracy of our presented OCR system, we used *Levenshtein edit distance* 5 between the output text and the ground-truth. Edit distance is calculated by computing the number of edit operations (insertions, substitutions and deletions) that are needed to convert a source string into the target string. The result is often normalized by the length of the target string to get the percentage error. Our system achieved an accuracy of 98% (error rate is 2.0%) compared to 88.94% (Ahmed et al. 2016), 94.85% (Ul-Hasan et al. 2013) (11.06 and 5.15% error rates) and 94.97% reported previously on the UPTI dataset.

**Fig. 9:**
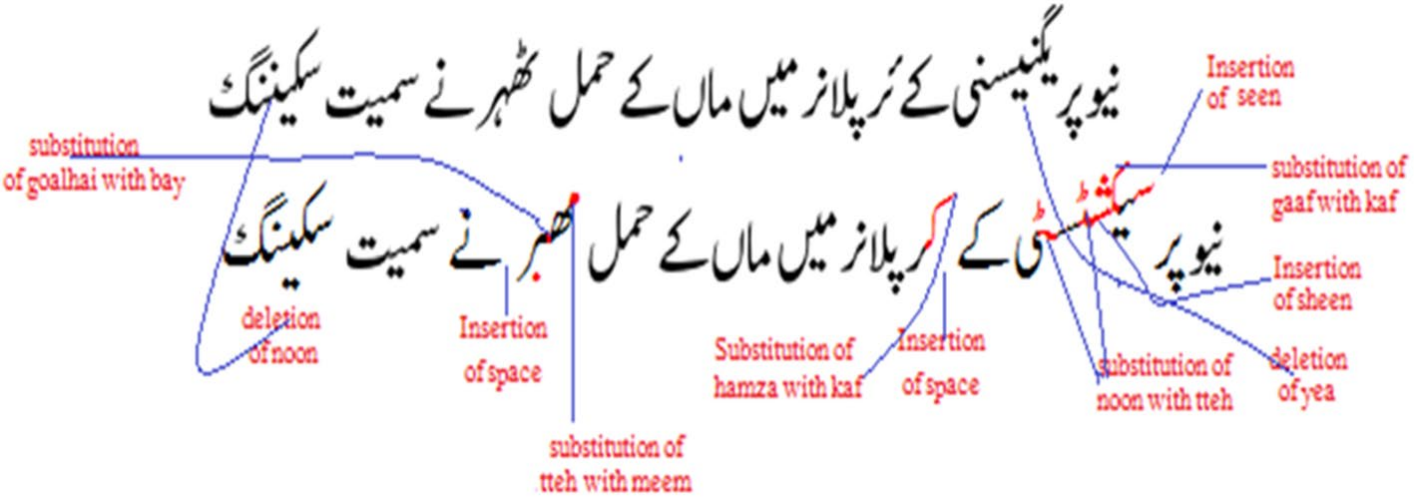
**a** An input image of Urdu Nasta’liq text-line, **b** output text produced by the MDLSTM recognition system: Illustration of deletion, insertion and substitution of characters after recognition are shown using *red*, *green* and *blue circles*

A sample input image and the corresponding OCR output text are shown in Fig. 9 with an illustration of insertion, deletion and substitution errors. A closer analysis of the results revealed that the most mis-classification errors originate from the recognition of “space” character. This issue is inherent in Nasta’liq script, because after each non-joiner character there is a space like gap (Naz et al. 2013a). However, it is not a “space” as it naturally occurs within a word when a non-joiner character is present at the initial or middle position of the word. Due to the compact nature of Nasta’liq script, spaces between words are not larger than the spaces withing ligatures of the same word that occur due to the above-mentioned characteristics of the non-joiner characters. Technically, it is even difficult for non-native speakers to distinguish this break from the regular “space” character. Thus, the MDLSTM model confuses the space character with the gap caused by non-joiner characters. The number of insertion and deletion errors for “space” are 304 and 279 respectively. If the errors related to spaces are ignored, the accuracy goes up to 99.82%. This indicates that our network is able to achieve near-perfect results on discriminating shapes of different characters. To further improve recognition of spaces and hence achieving better word segmentation, the use of language modeling could be explored (Durrani and Hussain 2010).

The second largest confusion is encountered with “noon” (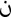) replaced by “teh” (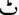). When we further investigated this confusion, the roots were again found in language specific characteristics. The base shape of “noon” (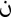) is the same as that of “teh” (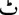) when it comes at Initial and at Middle position in a ligature/sub-word. The only difference is the special dot on the character “noon” (.) and the small tvain on the character “teh” (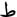), which are hard to discriminated due to their small sizes when used as a diacritic. Therefore, the “noon” character is confused by MDLSTM model with “teh” (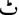) and vice versa, only if their position is initial or middle in a ligature.

**Fig. 10:**
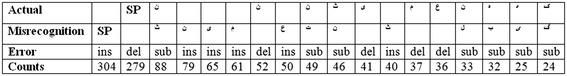
The confusion matrix showing the number of counts for most frequent mis-recognized characters on test set (*ins* insert, *subs* substitute, *del* delete)

In addition to the above-mentioned confusions, the diagonality factor of Urdu Nasta’liq is also examined. It is found that whenever the vertical overlap becomes dominant, especially when the adjacent characters partially overlap each other, the recognition becomes difficult. Another challenge is introduced by the presence of a large number of holes or diacritics/dots in close proximity to each other. As an example, see the second word shown in the Fig. 10, where the character “gaaf” (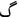) having two diagonal strokes like lines appears adjacent to the character “yea” (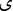) that has two dots below it. The occurence of these two challenges together resulted in substitution of “gaaf” (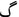) character with “kaf” (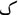) and insertion of a new character “seen” (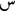). The most commonly confused characters along with their miss-recognized counts has been shown in Fig. 10.

**Fig. 11:**
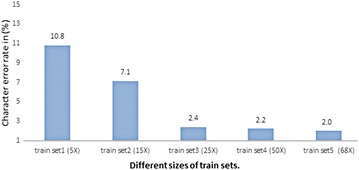
The recognition rate on 500 test text-lines on networks trained on different sizes of training sets

We have also conducted a number of experiments to analyze the sensitivity of recognition performance to the number of text-lines in the training set (see Fig. 11). We shuffled the text-lines in the training set and subsampled different subsets of sizes 500, 1500, 2500, 5000, and 6800 (the full sub-set). The test set consists of 500 test lines. To perform the evaluation of the above shuffled data subsets, we trained MDLSTM on each train data subset as a separate network and then the same test set is used to evaluate the performance of each trained network. The recognition results from each trained network showed that the error rates gradually decreased by increasing the number of text-lines in the training sets as shown in Fig. 11. Moreover, the experiments show that the error rate becomes stable from 2500 text-lines as there are insignificant changes in the error rate when the training set size is increased further to 5000 and 6800.

**Fig. 12:**
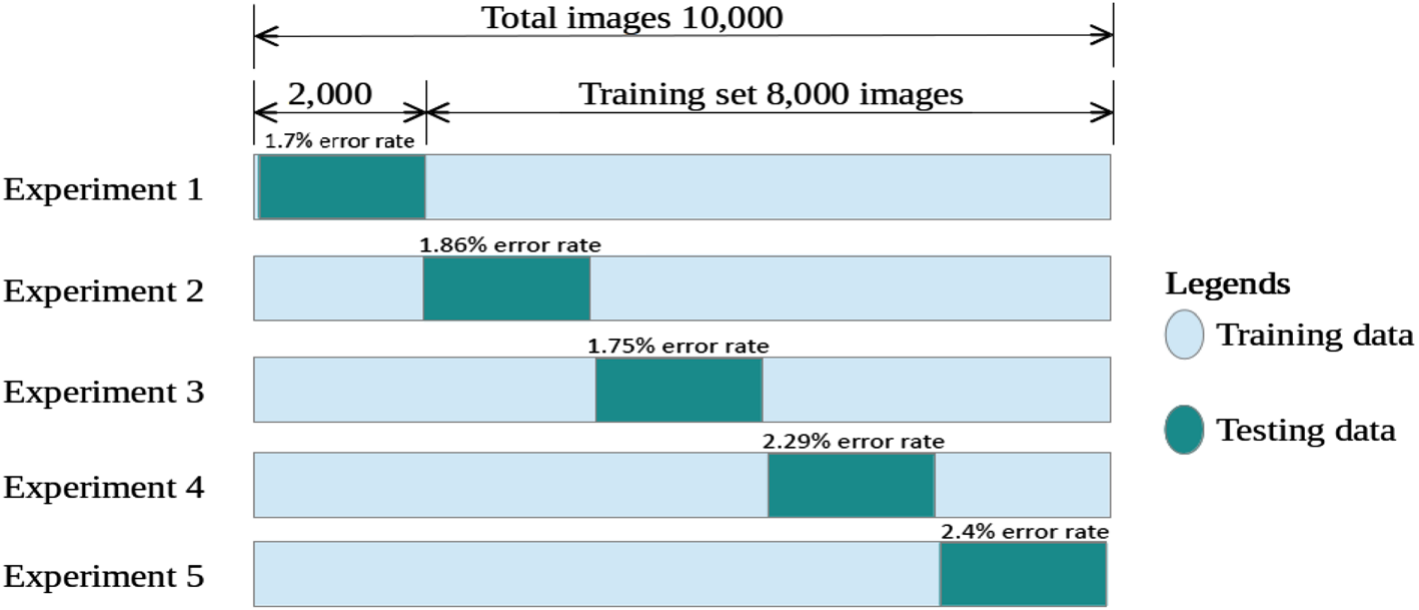
The overall split of the UPTI dataset for five-fold cross validation and error rate of each experiment

We also performed two types of experiments regarding cross validation. The purpose of cross validation is to analyse the impact of overall data samples on the learning of our proposed model. First, we conducted *k*-fold cross validation since it guarantees that each sample eventually become the part of training as well as testing sets. In addition, regarding the UPTI dataset, no standard training and testing splits are established in the literature. Therefore, by using *k*-fold cross validation we investigate any bias that might have been present in the previous study due to accidentally unbalanced partitioning of the data (e.g. the toughest images in terms of classification become part part of the training sets and hence do not appear in the test set). The UPTI dataset being used in this work contains 10,000 images. We are considering that our test set is the 20% of the entire dataset. This will make $k=5$, thus in the remaining sections this will be referred as 5-fold cross validation. The overall split of the dataset for 5-fold cross validation and the results obtained by 5-fold cross validation are shown in Fig. 12. Consistently low error rates on all partitions of the data indicate that our network indeed achieves good generalization and no training/test bias was present in the initial experiments.

In the second type of cross-validation, Repeated Random Sub-sampling Validation approach was applied. The dataset is shuffled and randomly divided into training, validation and testing data five times. Five sets of train-validation-test (68–16–16%) splits are made and then experiments are performed for each split. The recognition results are then averaged for all five splits. In these experiments, we got an average recognition rate of $98 \pm 0.25\%$, as shown in Table 5. The low standard deviation again indicates the stability of the recognition algorithm.

**Table 5:** Three types of analysis techniques employed for generalization of recognition error rates on UPTI dataset

Type of model validation technique	Error rate (%) of exp-1	Error rate (%) of exp-2	Error rate (%) of exp-3	Error rate (%) of exp-4	Error rate (%) of exp-5	Ave. error rate (%)
Train-set size based validation	10.8	7.1	2.4	2.2	2.0	–
Five-fold cross validation	1.7	1.86	1.75	2.29	2.4	2.0
Repeated random sub-sampling validation	2.32	1.36	2.0	2.03	2.29	2.0

A direct comparison of the proposed approach with the state-of-the-art Urdu text line recognition systems on UPTI dataset is given in Table 6. The proposed system provides significant improvement in the results as compared to the work done by Ul-Hasan et al. (2013) and Ahmed et al. (2016), both have employed BLSTM for Urdu text recognition. MDLSTM scans image in all four directions as compare to BLSTM which scans image only from right to left and left to right directions. The proposed system also provides significant improvements as compared to the work of Naz et al. (2015a, 2016a, b) who used statistical features with MDLSTM for recognition. The significant improvement of our results using raw pixels, shows that MDLSTM learns distinctive patterns very well from raw pixels. To the best of our knowledge, this is the first work based on MDLSTM using raw pixels for Urdu Nasta’liq text recognition. Our proposed system achieved a 2% error rate on Urdu Nasta’liq writing style despite its rich morphology, large alphabet size as well as variations in the shapes of the characters depending on the position of character occurrence in the word/sub-word.

**Table 6:** A comparison of the presented system on UPTI dataset with other techniques reported in the literature

Authors	Features	Approach	UPTI dataset	Ave. char. accuracy (%)
Ul-Hasan et al. (2013)	Pixels	BLSTM	46% train set	94.85
			34% validation set	
			20% test set	
Ahmed et al. (2016)	Pixels	BLSTM	46% train set	88.94
			44% validation set	
			10% test set	
Naz et al. (2015a)	Statistical features	MDLSTM	68% train set	94.97
			16% validation set	
			16% test set	
Proposed system	Pixels	MDLSTM	68% train set	98 ± 0.25
			16% validation set	
			16% test set	

## Conclusion

We presented an Urdu Nasta’liq text line recognition system using Multidimensional deep learning approach (MDLSTM). The proposed approach is particularly suitable due to the diagonal nature of the script. Our results demonstrate that the presented system out-performed state-of-the-art approaches based on Bidirectional LSTM networks. We also show that automated feature extraction using raw pixels as input to MDLSTM classifier achieved better results than manually designed statistical features. Results of our approach on publicly available UPTI dataset show an over 50% reduction in error rate as compared to state-of-the-art systems.

## Footnotes

1
www.abbyy.com.

2
www.MeOCRe.com.

3
http://www.jocr-offline-offline-japanese-english-dictionary-and-camera-recognition-ios.html.

4
http://jang.com.pk/.

5
http://en.wikipedia.org/wiki/Levenshtein_distance.
